# Root Canal Therapy of a Mandibular First Molar with Five Root Canals: A Case Report

**Published:** 2007-10-02

**Authors:** Mohammad Frough Reyhani, Saeed Rahimi, Shahriar Shahi

**Affiliations:** *1. Department of Endodontics, Dental School, Tabriz University of Medical Sciences, Tabriz, Iran*

**Keywords:** Case Report, Mandibular First Molar, Root Canal Therapy

## Abstract

A mandibular first molar requiring root canal therapy was found with five canals, three mesial canals, and two distal canals. Initially, four canals (mesiobuccal, mesiolingual, distobuccal, and distolingual) were identified. The mesiobuccal and mesiolingual canals were found in their normal locations, and a fifth canal was noted between these two. This case demonstrates a rare anatomical configuration and supplements previous reports of the existence of such configurations in mandibular first molars.

## INTRODUCTION

The treatment of the entire root canal system is essential to maximize the possibility of obtaining success in the endodontic therapy. It is necessary for the clinician to have a thorough knowledge of the dental anatomy as well as of its variations. The mandibular first molar is the first posterior tooth that erupts and is the tooth that most often requires root canal treatment This tooth usually has two roots, but, occasionally, it has three (1), with two or three canals in the mesial root and one, two, or three canals in the distal root. There are numerous cases in the literature concerning the unusual anatomy of the mandibular first molar.

The presence of a third canal in the mesial root of mandibular first molars has been reported to have an incidence rate of 1 to 15% (2). This additional canal may have a separate foramen, or join apically with either the mesiobuccal or mesiolingual canal (2). In 1985, Martinez-Berna and Badanelli (3) reported that 1.5% of the 1418 teeth had three canals in the mesial root. In 1985 and 1989, Fabra-Campos (4,5) displayed middle mesial canal in 2.1% and 2.6% of the teeth, respectively. In recent studies it has been shown that the prevalence of three canals in mesial root of mandibular first molar teeth was 3.3% in South Asian Pakistanis (6) and 2% in Sudanese population (7).

A radiographic study of extracted teeth reported mandibular first molars with three mesial canals in 13.3% of specimens, four mesial canals in 3.3% of specimens, and three distal canals in 1.7% of specimens (8). Although the sample size in that study was small, it emphasized the possibility of additional canals. The present report describes root canal treatment in a right mandibular first molar with five canals, three of which are located in mesial root.

## CASE REPORT

**Figure 1:**
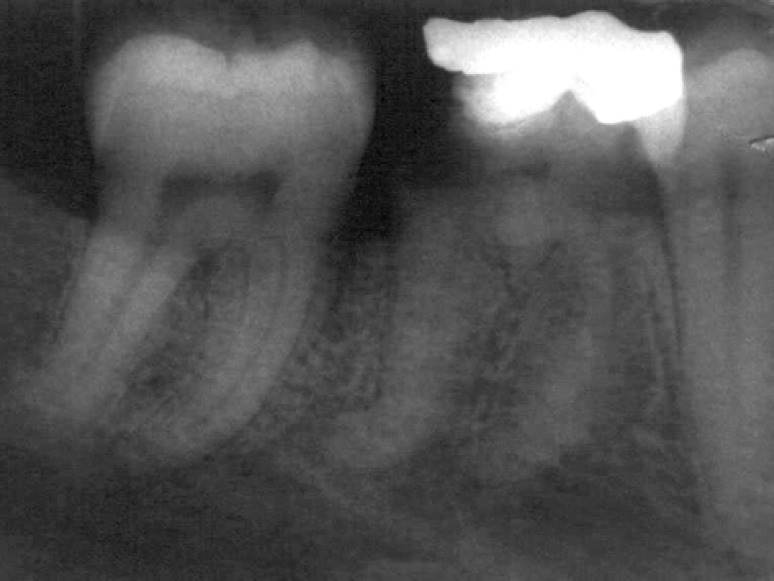
Preoperative radiograph

A 43-year-old male patient was referred to the dental clinic for endodontic treatment of tooth #30. His medical history was found to be noncontributory. The patient’s chief complaint was pain in his lower right back teeth region. Clinical examinations revealed that tooth had deep disto-occlusal caries. The tooth was tender to vertical percussion but not to palpation and the periodontal condition of the tooth was normal and no pocketing was observed. On sensitivity tests, using heated gutta-percha stick, ice stick, and electric pulp tester, there was a severe, long-lasting painful response. The reason of his pain was diagnosed to be irreversible pulpitis in his mandibular right first molar. Aberrant features of the mesial canals were observed on the preoperative radiograph, suggestive of unusual morphology of the mesial canal system (Figure 1). The right inferior alveolar nerve was anaesthetized using 2% Lidocaine with 1:80000 adrenaline (Daroupakhsh, Tehran, Iran). After rubber dam placement, the old filling material and all carious tissue were removed and the access cavity prepared. After an adequate access preparation was made, two mesial and two distal canal orifices were found.

**Figure 2:**
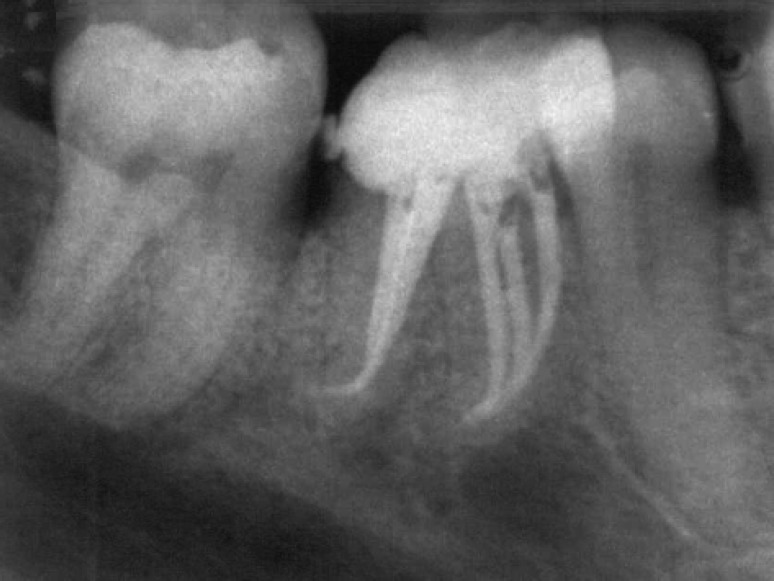
Postoperative radiograph

Examining the fissure connecting the two mesial canals carefully, the tip of a # 08 file could be inserted into a spot located in the middle of the distance between the two mesial canals. The middle mesial canal originated as a separate orifice but joined to the mesiolingual canal in the apical third of the canal. The two distal canals converged to a common apical foramen. The root canals were cleaned and shaped using flexofile files (Maillefer, Switzerland) and Gates Glidden drills #2, #3, and #4 (Mani, Japan) with passive step-back technique. Apical preparations in the mesial and distal canals were enlarged to a master file size of 30. The root canals were copiously irrigated with 1% sodium hypochlorite solution. Before obturation, master points were seated to test their suitability to canals. Then, the canals were dried with sterilized paper points and obturated with AH26 sealer (De Trey, Dentsply, Konstanz, Germany) and gutta-percha using a lateral compaction technique (Figure 2).

After removal of excess gutta-percha cones, a sterile cotton pellet was put into the pulp chamber and the access sealed with Cavit (ESPE, Seefeld, Germany).

## DISCUSSION

Based on the literature and this clinical case, it is evident that knowledge of the anatomical variations of the mandibular molars is extremely important for the success of endodontic treatment. According to Cohen and Burns (9), canals are often not treated because they are not located. Once endodontic treatment has been initiated, proper access cavity preparation is a basic prerequisite for the investigation and successful detection of all root canal orifices. The use of microscope enables dentists to locate and treat extra canals confidently (10).

The root canal anatomy of the mandibular first molar can be aberrant. Clinicians must be aware of the finding that the presence of a third canal in the mesial root of the mandibular first molars has been reported to have an incidence rate of 1% to 15%. In the study of Pomeranz *et al.* (11), the additional canal may be classified as (a) an independent canal, which originates in a separate orifice and terminates as a separate foramen, (b) a confluent canal, that originates as a separate orifice but is apically joined to the mesiobuccal or mesiolingual canal, and (c) a fin, when the instrument can pass freely between the mesiobuccal or mesiolingual canals and the middle mesial canal during cleaning and shaping.

In order to treat a mandibular molar with 5 canals, it is necessary to check their clinical and radiographic anatomy. One should perform a thorough examination of the pulp chamber to ensure a more accurate orifice location, and then completely clean all canals. This increases the chance of finding an extra canal and the long-term success rate of endodontic therapy.

In this case, we found that the middle mesial canal originated as a separate orifice but joined the one-third apical of the mesio-lingual canal. According to Pomeranz *et al*. classification (11), the middle mesial canal is classified as confluent. In the past, many studies have reached the conclusion that failure in tracing and cleaning extra canals in any root decreases the long-term prognosis of endodontic treatment. All these extra canals, if any, should be found if possible.

## CONCLUSION

This case provides evidence that mesial roots in mandibular first molars can contain three canals. Although the incidence of a middle mesial canal is not high, it is important to take this variation into consideration during root canal therapy of mandibular molars in order to ensure long term treatment success.
